# Mapping the evolution of acne research based on 100 top-cited articles: A bibliometric analysis of trends and hotspots from 2014 to 2023

**DOI:** 10.1097/MD.0000000000037657

**Published:** 2024-05-24

**Authors:** Lu Wei, Buxin Zhang, Li Wang, Aimin Liu

**Affiliations:** a Henan University of Chinese Medicine, Zhengzhou, China; b Department of Dermatology, Henan Province Hospital of Traditional Chinese Medicine, The Second Affiliated Hospital of Henan University of Chinese Medicine, Zhengzhou, China.

**Keywords:** acne, bibliometric analysis, hotspot, microbiota dysbiosis, western diet

## Abstract

**Background:**

Acne is a prevalent inflammatory condition of the pilosebaceous unit, which seriously affects the appearance and mental health of patients. Bibliometrics is the statistical analysis of academic literature in a certain field. We aimed to characterize the 100 most cited articles on acne from a bibliometric perspective, as well as explore the frontier hotspots and trends of acne.

**Methods:**

A search was conducted on the Web of Science database on August 8, 2023. we employed the terms “acne,” “acne Vulgaris,” and “common acne” in our search. The top 100 articles with the most citations throughout the time frame of 2014 to 2023 were discovered and assessed. The visualization study was carried out using bibliometric tools such as CiteSpace 6.2.R4, VOSviewer 1.6.18, and MapChart.

**Results:**

The top 100 most cited articles were published between 2014 and 2021, originated from a diverse range of 48 countries, with a predominant focus on the United States of America (USA) and Germany. The top 100 papers were cited between 50 and 712 times. Dreno B, from Nantes University, was the most frequently nominated author. With 12 papers, the *Journal of the European Academy of Dermatology and Venereology* contributed the most to the top 100 list. Alongside the term “acne”, the following terms or phrases were observed frequency in the top 100 articles, *Cutibacterium* acnes, sebaceous, western diet, antibiotic resistance, staphylococcus-epidermidis, insulinlike growth factor 1, benzoyl peroxide, and polyunsaturated fatty acids. Alongside the term “acne”, terms or phrases such as *Cutibacterium* acnes, sebaceous, western diet, antibiotic resistance, staphylococcus-epidermidis, insulinlike growth factor 1, benzoyl peroxide, and polyunsaturated fatty acids, etc also have a high frequency in the top 100 articles.

**Conclusion:**

This analysis summarizes the shifting trends of acne research over the last decades. Research on acne is currently flourishing. The related pathogenesis and therapeutic strategies have been the focus of current research and developmental trends in future research.

## 1. Introduction

Acne is a prevalent inflammatory condition of the pilosebaceous unit, predominantly affecting the face and trunk, with a global prevalence of roughly 9%.^[1]^ There is evidence that severe acne is associated with increased incidence rate of anxiety, depression, and suicidal ideation.^[2]^ The current primary treatments for acne, such as benzoyl peroxide, topical retinoids, and either topical or oral antibiotics, focus on 1 or 2 stages in the development of the condition.^[3]^ However, the rising global prevalence of antibiotic resistance has raised concerns regarding acne treatment. The aforementioned fact has served as a driving force for the exploration of novel drugs aimed at delaying the progression of acne while causing fewer side effects.

The quantification of citations serves as a bibliometric indicator that indirectly reflects the quality, influence, production, and status of a scholarly work.^[4]^ Bibliometric analysis enables the identification of highly cited works, hence allowing for the characterization of scientific production within a certain topic of interest.^[5]^ The utilization of bibliometric parameters for the identification and characterization of scientific production has the potential to enhance comprehension regarding the progress and direction of research on acne. This study sought to analyze the top 100 most significant articles over the preceding decade through a bibliometric approach to characterize the research status and trends within this field.

## 2. Materials and methods

### 2.1. Database and search methodology

The study employed the Web of Science Core Collection to conduct the literature search. The search formula was set to TS = (“acne”) OR TS= (“acne vulgaris”) OR TS= (“common acne”). The selected publication period spanned from January 1, 2014 through August 11, 2023, without any restrictions on language, document categories, or research approach. The findings were arranged in descending order based on the frequency of citations recorded in the WOS. Two scholars picked articles independently until the 100th most referenced paper was discovered. Disagreements were settled through discussion.

### 2.2. Data extraction

Our analytic scope comprised studies with acne as a primary or secondary focus. The essential information such as titles, authors, countries, etc, was extracted from the WOS core database and imported into an Excel spreadsheet. In the case of a tie, the citation density (the number of citations per year) was used to determine the order. The journal impact factors (IF) were obtained from the 2022 edition of the Journal Citation Reports. Keywords with similar meanings but distinct styles were standardized: “*Propionibacterium acnes*” was replaced by “*Cutibacterium acne*s (formerly identified as *Propionibacterium acnes*),” “il-17” was replaced by “interleukin-17,” “acne-vulgaris” was replaced by “acne vulgaris.” The utilization of the average citations per year (ACY) score aimed to reduce the influence of temporal bias, hence enabling a more equitable evaluation of junior academics. ACY = citation times/(2023-publication year + 1).

### 2.3. Bibliometric analysis

MapChart was utilized to visually depict the distribution of publications across different countries and continents. The chromatic intensity exhibits fluctuations contingent upon the existence or nonexistence of acne-related articles possessing a substantial quantity of citations across different countries. Countries with a greater degree of color intensity had more acne-related citations in the scientific literature. The VOSviewer software was employed to generate country co-authorship analysis, author co-occurrence cluster maps, and keywords co-occurrence analysis. CiteSpace was utilized to conduct collaboration network analysis of institutions. In the network visualization maps, different nodes represent different elements such as countries, authors or keywords. The size of the nodes reflects the number of outputs, citations or occurrences and the color indicates different clusters or average appearing year of these elements. Lines were made to show the relationship between elements, whereas the connections between nodes signify associations of co-occurrence.

## 3. Results

A total of 10, 474 documents were obtained from the Web of Science Core Collection databases for the period spanning from 2014 to 2023. The top 100 highly cited articles are arranged in descending order according to their overall citation count (Table S1, Supplemental Digital Content, http://links.lww.com/MD/M32). For the study types of the T100 articles on acne, 64 were articles, 34 were reviews, 1 was comment, and 1 was others.

### 3.1. Publication year

**Figure 1.**
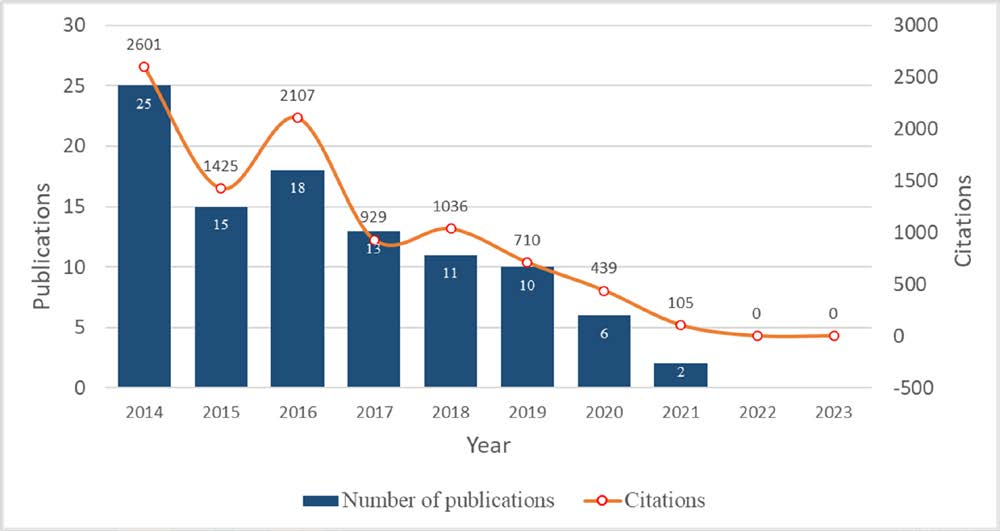
Number of top-cited publications from 2014 to 2023.

Publication years for the T100 articles range from 2014 to 2021. Figure 1 depicts the yearly quantity of papers that were published over the last decade. In general, the publications that have received significant citations show a fluctuating downward trend. The year that yielded the most quantity of high-impact publications was 2014 (n = 25), followed by 2016 (n = 18), and 2015 (n = 15).

### 3.2. Citations

The T100 publications were cited 9352 times, with individual citations ranging from 50 to 712. The median citation count for these papers was 70.5. Approximately 25% of the articles (n = 26) garnered over 100 citations, while a mere 4 articles obtained citations above 200. In the year 2014, the number of citations reached its peak with 2601 citations. The paper that has garnered the most citations is authored by Zaenglein AL et al^[6]^ and published in *Journal of the American Academy of Dermatology* in 2016 (Table S1, Supplemental Digital Content, http://links.lww.com/MD/M32). Additionally, this article has the highest ACY. The most recent article was authored by Eichenfield DZ et al^[7]^ and published in November 2021 in the *Journal of the American Medical Association*.

### 3.3. Contributions of countries

**Figure 2.**
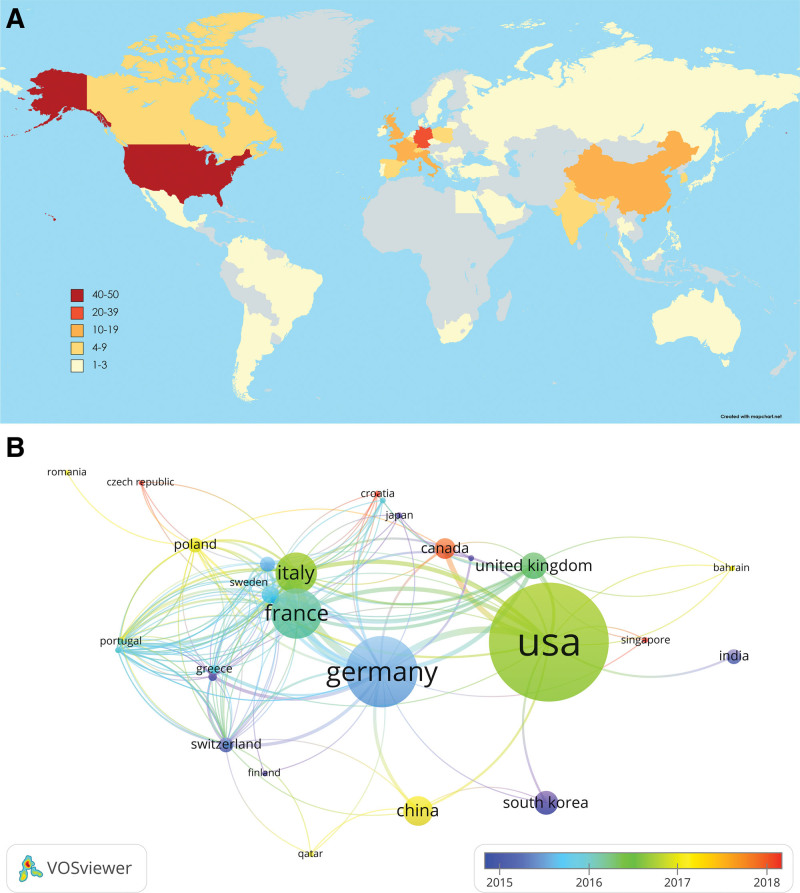
(A) Number of top-cited publications by countries. (B) Map of cooperation network between countries.

The T100 most-cited papers have authors from 48 different countries. It is noteworthy that 23 countries/regions surpassed the threshold of having more than 3 articles, as shown in Figure 2A. A collaborative network was established among countries through VOSviewer (Fig. 2B). The USA is the foremost contributor, closely followed by Germany, France, Canada, and China. The USA also is the country with the biggest number of citations and the highest total link strength among countries, and it is the country with the strongest collaborative linkages with Germany, France, and Italy. In addition, we also find that countries such as Canada, Singapore, and Croatia have actively engaged in international research collaborations, emerging as influential contributors in the field of acne research in recent years.

### 3.4. Contributions of institutions

**Figure 3.**
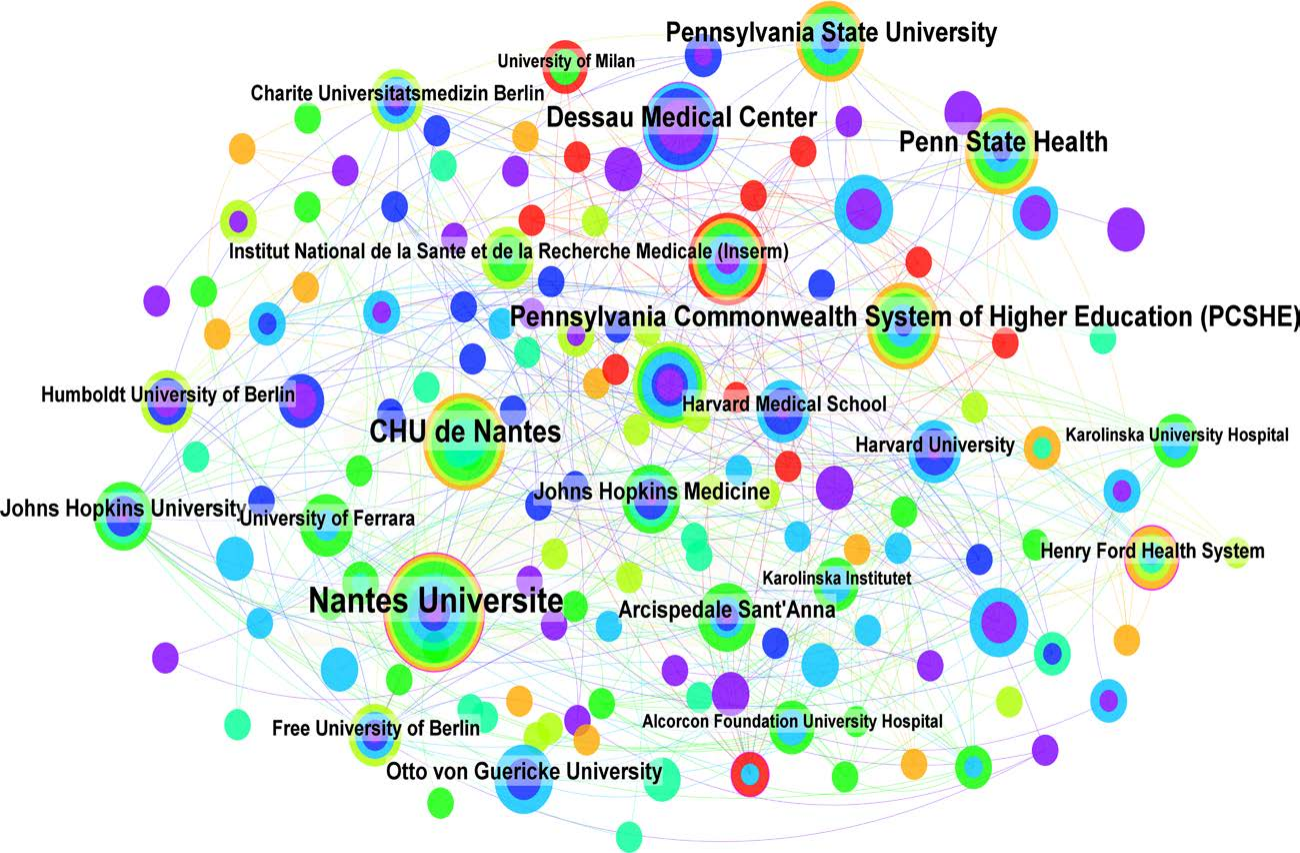
Network visualization map of institution co-authorship analysis.

A grand total of 418 academic institutions made contributions to the T100 articles. The top institution is Nantes Universite, which has published 15 articles with a total of 1651 citations. The University of California System ranked second (n = 11) and CHU de Nantes ranked third (n = 10), both accumulating over 1100 citations. In its collaborative network, Nantes Universite engages in collaborative partnerships with 43 distinct institutions. Of these, it maintains the closest collaboration with Penn State Health, Pennsylvania State University, and Johns Hopkins Medicine (Fig. 3).

### 3.5. Major contributing authors

**Figure 4.**
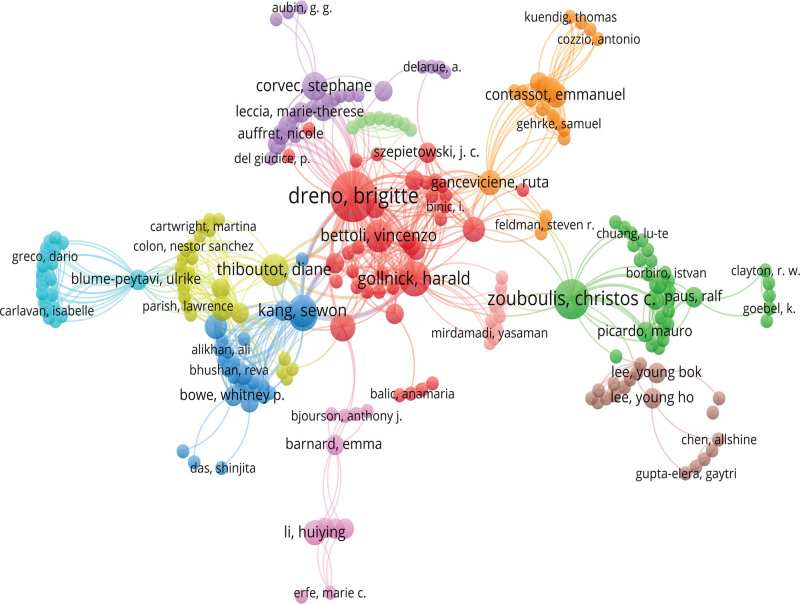
Author cluster map generated by VOSviewer.

There were 537 authors involved in the top 100 most-cited articles. Table 1 displays the data indicating that a total of 7 writers made significant contributions by producing more than 5 highly referenced publications. Dreno B from Nantes Universite was the top contributors with 13 articles, followed by Zouboulis CC from Brandenburg Medical School Theodore Fontane (n = 8), and Gollnick H from Guericke University (n = 6). The most cited author was Dreno B (n = 1329). Besides, Dreno B has the highest total link strength (81). The article by Leyden JJ had the highest average number of citations (n = 200.8). Figure 4 illustrates the network of author collaborations, with 472 authors having cooperative relationships. Different colored clusters represent different teams of authors, forming 11 teams of authors. Among them, Dreno B, Zouboulis CC, and Thiboutot D cooperated the most with other authors. Dreno B, Gollnick H, and Bettoli V have collectively authored 5 research articles, indicating a consistent and enduring partnership in their scholarly endeavors.

**Table 1:** Top 10 authors in the top 100 most-cited publications.

Author	TP	TC	CPP	TLS	Institution	Country
Dreno B	13	1379	106.08	81	Nantes Universite	France
Zouboulis CC	8	887	110.88	56	Brandenburg Medical School Theodore Fontane	Germany
Gollnick H	6	528	88.00	57	Guericke University	Germany
Leyden JJ	5	1004	200.80	38	University of Pennsylvania	USA
Kang S	5	997	199.40	41	Korea Research Institute of Bioscience & Biotechnology (KRIBB)	South Korea
Bettoli V	5	439	87.80	50	University of Ferrara	Italy
Thiboutot DM	5	353	70.60	40	Pennsylvania Commonwealth System of Higher Education (PCSHE)	USA
Corvec S	4	466	116.50	16	CHU de Nantes	France
Kim J	4	371	92.75	24	University of California Los Angeles	USA
Layton AM	4	346	86.50	32	University of York - UK	UK

CPP = number of citations per publication, TC = total citations, TLS = total link strength, TP = total publications, UK = United Kingdom, USA = United States of America.

### 3.6. Distribution of journals

The T100 papers were published in 47 journals. Information about the leading 10 journals that have published the greatest quantity of T100 papers is provided in Table 2. The *Journal of the European Academy of Dermatology and Venereology* exhibits the greatest quantity of articles in the list (n = 12), followed closely by *Journal of Investigative Dermatology* (n = 10) and *Journal of the American Academy of Dermatology* (n = 7). Besides, the *Journal of the European Academy of Dermatology and Venereology* was the most frequently cited journal (n = 1369). According to the Journal Citation Reports categories, it is seen that 7 of the top 10 journals are classified under the Q1 category. Among them, *Journal of the American Academy of Dermatology* has the highest IF (13.8).

**Table 2:** Top 10 journals with the most publications.

Ranking	Sources Title	TP	TC	CPP	JCR	IF 2022
1	*Journal of the European Academy of Dermatology and Venereology*	12	1369	114.08	Q1	9.2
2	*Journal of Investigative Dermatology*	10	895	89.5	Q1	6.5
3	*Journal of the American Academy of Dermatology*	7	1180	168.57	Q1	13.8
4	*British Journal of Dermatology*	5	553	110.6	Q1	10.3
5	*JAMA Dermatology*	5	360	72	Q1	10.9
6	*American Journal of Clinical Dermatology*	5	327	65.4	Q1	7.3
7	*Clinics in Dermatology*	4	250	62.5	Q2	2.7
8	*Journal of Cosmetic Dermatology*	4	250	62.5	Q3	2.3
9	*Experimental Dermatology*	3	207	69	Q1	3.6
10*	*Archives of Dermatological Research*	3	196	65.33	Q2	3
10*	*Dermatologic Therapy*	3	173	57.67	Q1	3.6

CPP = number of citations per publication, IF = impact factor, JCR = Journal Citation Reports, TC = total citations, TP = total publications.

### 3.7. Co-occurrence analysis of keywords

**Figure 5.**
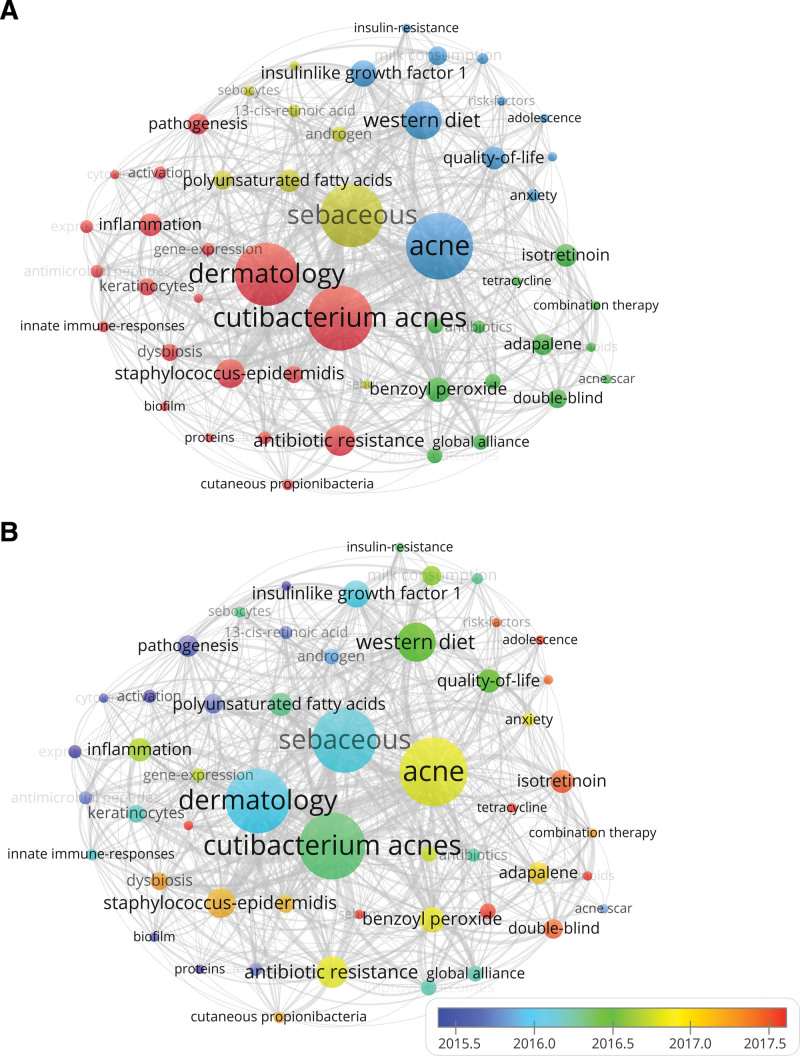
Keywords analysis for research of acne. (A) Clustering co-occurrence map of keywords. (B) Distribution of keywords based on the average time of appearance.

A total of 629 keywords were encompassed in this study. Table 3 lists the 30 most frequently occurrences keywords. Alongside the search terms, the most commonly researched keywords were *Cutibacterium acnes* (n = 34), dermatology (n = 33), sebaceous (n = 33), western diet (n = 20), and antibiotic resistance (n = 16). The VOSviewer software was utilized to generate the keyword co-occurrence network (Fig. 5A). Clustering effectively reflects current research hotspots. Figure 5A demonstrates how keywords are grouped into 11 clusters, each represented by a different color. The keywords can be clustered into 4 distinct groups, each represented by a different color (red, blue, green, yellow). The red cluster mainly represents *Cutibacterium acnes* (including dermatology, staphylococcus-epidermidis, antibiotic resistance). The blue cluster is primarily related to acne (including western diet, insulin like growth factor 1, milk consumption, quality-of-life, and anxiety). The green clusters focused on benzoyl peroxide (including isotretinoin, adapalene, and double-blind). The yellow clusters predominantly associated with sebaceous (including polyunsaturated fatty acids, androgen, and sebocytes). These high-frequency keywords reflect that the research on acne is mainly in *Cutibacterium acnes*, sebaceous, western diet, polyunsaturated fatty acids, antibiotic resistance, and benzoyl peroxide. In general, we can see that research on acne focuses on 2 directions, namely the exploration of its related pathogenesis and treatment strategies. The color depth indicates that the distribution of keywords followed a chronological order (Fig. 5B).

**Table 3:** Top 30 most frequent occurrences terms in the titles and abstracts.

Ranking	Keyword	Occurrences	Total link strength
1	Acne	35	267
2	*Cutibacterium acnes*	34	281
3	Dermatology	33	231
4	Sebaceous	33	214
5	Western diet	20	148
6	Antibiotic resistance	16	132
7	Staphylococcus-epidermidis	15	126
8	Insulin-like growth factor 1	14	109
9	Benzoyl peroxide	13	114
10	Polyunsaturated fatty acids	12	94
11	Inflammation	12	92
12	Quality-of-life	12	90
13	Isotretinoin	12	83
14	Adapalene	11	75
15	Pathogenesis	11	75
16	Toll-like receptors	10	78
17	Double-blind	10	63
18	Milk consumption	10	63
19	Microbiome	9	99
20	Dysbiosis	9	82
21	Keratinocytes	9	76
22	Androgen	8	65
23	Global alliance	8	61
24	Improve outcomes	8	61
25	Management	8	36
26	Efficacy	8	35
27	Antibiotics	7	68
28	Gene-expression	7	55
29	Antimicrobial peptides	7	52
30	Anxiety	7	51

## 4. Discussions

### 4.1. Basic information analysis

The studies that receive the highest number of citations within a specific topic are commonly regarded as seminal works, possessing significant reference value for subsequent study due to their pioneering contributions. From the list, the earliest article on the list was published in 2014, while the most recent was in 2021. The years that produced a very substantial number of papers with significant influence were 2014, 2016, and 2015. The USA was the country with the highest quantity of scholarly articles and the most citations, so signifying its preeminent influence in this particular field. Meanwhile, the rising incidence of acne in the USA also serves as a driving force for them to pursue approaches for acne therapy.^[8]^ The USA, Germany, and France were the most active collaborators and played an important role in international cooperation. The institution with a remarkable publication count (n = 15) and an impressive average citation frequency (n = 110.07) was Nantes Universite, reflecting its prominent standing within the domain of acne research.

The top-cited studies were predominantly published in reputable dermatology journals such as the *Journal of the European Academy of Dermatology and Venereology*. This observation implies that these journals have a tendency to publish articles of acne. Meanwhile, it is evident that a majority of these journals possess a substantial IF. This finding further reinforces the established notion that journals with high IF frequently publish high-quality studies, hence sustaining their own IF.^[9]^

An article’s quality can be gauged by its citation count.^[10]^ Scholarly articles that have garnered more than 100 citations are commonly acknowledged as seminal works or classics. It is seen that a total of 26 articles garnered over 100 citations, making them true classics. The other articles garnered a minimum of 50 citations. Typically, older articles have a tendency to accrue citations as time progresses, eventually becoming into references. However, the article with the most cited publication on the list was published in 2016 and was regarded as a foundational study in the area.^[6]^ This evidence-based guideline is focused on addressing important clinical questions that occur in acne management. Despite their relatively lower citation numbers, more recent articles offer fresh avenues for exploration within the relevant topic. The latest article on the list summarized the key points of diagnosing and treating acne.^[7]^

Dreno B from Nantes Universite emerges as the most prolific and cited researcher overall, followed by Zouboulis CC from Brandenburg Medical School Theodore Fontane. However, when considering the mean number of citations per article, Leyden JJ ranks at the top of the list. Dreno B has emerged as the leading contributor inside the cluster, with the largest number of research publications (n = 39) and a notably high link strength (total link strength = 81). However, it is worth noting that Dreno B (n citation = 106.08) does not own the highest citation count among the authors. Dreno B focuses on investigating the diversity of skin microbiota and asserts that the loss of the skin microbial diversity contributes to the development of acne.^[11]^ In addition, he has been actively engaged in revision of acne treatment guidelines.^[12]^ Zouboulis CC is dedicated to investigating the pathophysiology of acne and emphasizes that the ratio of skin surface lipids and alterations in lipid composition are the primary factors contributing to the development of acne inflammation.^[13]^

### 4.2. Research hotspots and fronts

Through the analysis of high-frequency keywords, the top 100 most-cited articles included a wide variety of acne-related themes. By employing the methodology of keyword clustering and trend theme analysis, we outline the existing hotspots and potential developments direction of acne.

#### 4.2.1. Pathogenesis

According to metagenomic sequencing, it has been determined that the commensal *Cutibacterium acnes* is the predominant resident local bacteria detected in the healthy skin’s sebaceous unit, followed by *Corynebacterium* and *Staphylococcus*.^[14]^ The overproduction of IL-17 and activation of Th17 cells have been demonstrated to underlie the development of acne.^[15]^ *Cutibacterium acnes* phylotype IA1 via Toll-like receptor 2-dependent signaling, to trigger the production of IL-6, IL-8, transforming growth factor-β, IL-1β and induces CD4 lymphocytes differentiate into Th17 lymphocytes.^[16]^ The virulence profile of *Cutibacterium acnes* is associated with alterations in the microenvironment of the bacterium located within the pilosebaceous follicle. These alterations include an excessive production of sebum (hyperseborrhea), changes in the composition of sebum content (dysseborrhea), and the formation of biofilms.^[17]^ Biofilms produced by *Cutibacterium acnes* can enhance their resistance to antimicrobial treatments. One significant drawback associated with conventional topical antibiotic treatment is its tendency to cause cutaneous dysbiosis and promote the development of antimicrobial resistance. The microbiome is known to exert a substantial influence on acne development. The primarily cause of acne is local dysbiosis, rather than bacterial hyperproliferation.^[18]^ Therefore, the forthcoming therapies would no longer eradicate *Cutibacterium acnes*, but rather preserve the equilibrium of the skin microbiota, achieve tissue homeostasis, particularly the recovery of *Cutibacterium acnes* phylotype diversity.^[11]^ The involvement of the gut microbiota in the pathogenesis of acne has also been suggested. The interaction between metabolites generated by gut microbiota and mammalian target of rapamycin (mTOR) signaling pathways has been demonstrated to influence intestinal microbiota composition and have consequences for acne pathogenesis.^[19]^

Western dietary patterns are distinguished by a notable elevation in glycemic load and an increase in milk and dairy consumption. According to the literature, there is evidence suggesting a correlation between the consumption of milk, sugary drinks, fatty and sugary goods, and the occurrence of acne.^[20]^ The consumption of milk is associated with the increase level of insulin-like growth factor 1.^[21]^ Consuming a meal with a high-glycemic-load leads to elevated levels of insulin and insulin-like growth factor 1 in the bloodstream. This, in turn, triggers the processes of lipogenesis and proliferation of keratinocytes and sebocytes. Additionally, it promotes the synthesis of androgens and enhances the activation of mTORC1.^[22]^ The mTORC1 pathway have a significant impact on the pathogenesis of acne by regulating sebaceous gland hyperproliferation, lipid synthesis, and keratinocyte hyperplasia.^[23]^ Acne patients commonly exhibit increased regulatory activity of mTORC1, which is frequently associated with type 2 diabetes, insulin resistance, and obesity.^[24]^ Additionally, it was shown that a low-glycemic load diet resulted in an increased proportion of saturated to monounsaturated fatty acids in skin surface triglycerides, as well as a decrease in the occurrence of acne lesions.^[25]^ The increase in consumption of trans-fats and saturated fats is positively correlated with the severity of acne.^[26]^ The introduction of supplement containing ω-3 fatty acids or γ-linoleic acid have been shown to significantly reduce both inflammatory and noninflammatory acne lesions.^[27]^

#### 4.2.2. Treatment

First-line treatments for acne include topical applications of retinoids, benzoyl peroxide, azelaic acid, and/or combinations of these. The US Food and Drug Administration has granted approval for the utilization of 4 topical retinoids in acne therapy. These retinoids include tretinoin, adapalene, tazarotene, and trifarotene. Oral isotretinoin is the only therapeutic approach that directly or indirectly addresses all currently recognized pathogenic factors associated with acne.^[28]^ Tetracyclines are widely utilized oral antibiotics that effectively decrease *Cutibacterium acnes* and exhibit anti-inflammatory properties. The use of oral antibiotics is a frequently employed approach to achieve relatively rapid control of moderate to severe inflammatory acne. Typically, oral antibiotics are utilized in conjunction with a topical retinoid and benzoyl peroxide. However, antibiotic-induced antimicrobial resistance is becoming increasingly significant on a worldwide scale. The present recommendations in all acne guidelines strive to minimize the utilization of antibiotics for acne treatment. Consequently, there is growing interest in the utilization of antimicrobial peptides and agents that regulate populations of *Cutibacterium acnes*, including probiotic preparations and gels that degrade biofilm matrices. In addition to mild comedolytic and anti-inflammatory properties, benzoyl peroxide restricts the development of bacterial resistance to topical and oral antibiotics, and has higher efficacy than topical antibiotics alone.^[29]^

Öncü I et al^[30]^ conducted an assessment of somatosensory amplification, anxiety, and depression levels among acne patients. The researchers determined that there exists a noteworthy association between somatosensory amplification, depression, and health anxiety among individuals with acne. They argue that an interdisciplinary strategy that integrates both psychiatry and dermatology would be advantageous for the treatment of acne and the overall management of patients.

### 4.3. Limitations

Our study also has several limitations. First, our study exclusively relied on the WoSCC database as the primary source of data, which may result in the omission of pertinent papers available in other databases such as Scopus and Pubmed. Among these databases, WoSCC is considered a dependable and reputable source for accessing international peer-reviewed papers. Second, it is important to acknowledge that earlier researches are likely to have a larger frequency of citations compared to more recently published ones due to the time factor. However, it should be highlighted that the academic effect of earlier studies may not necessarily be stronger than that of later ones. Therefore, it is possible that certain significant publications, which have emerged recently, could be excluded due to insufficient time for the accumulation of citations. Third, the extent to which citation counts accurately reflect academic influence is a topic of ongoing debate, despite their widespread usage as a reference index. Therefore, it is essential to assess the whole influence of a study comprehensively.

## 5. Conclusions

A bibliometric analysis was performed in this study to identify the top 100 articles on acne published in the past decade. When looking at total quantity of T100 articles, the USA clearly dominates. The *Journal of the European Academy of Dermatology and Venereology* experienced the highest number of T100 papers published during the years 2014 and 2023. Dreno B has made substantial contributions to the domain of acne research. The primary academic emphasis lies in the examination of the pathophysiological mechanisms and therapeutic strategies of acne, with particular attention given to the involvement of microbiota dysbiosis and western diet in the pathogenesis of acne. Further comprehensive investigations in this field will facilitate the identification of other possible therapeutic targets.

## Acknowledgments

The authors appreciate the publications included in this study.

## Author contributions

**Conceptualization:** Aimin Liu.

**Data curation:** Lu Wei, Buxin Zhang.

**Formal analysis:** Lu Wei, Li Wang.

**Methodology:** Lu Wei, Buxin Zhang.

**Software:** Buxin Zhang.

**Writing – original draft:** Lu Wei.

**Writing – review & editing:** Aimin Liu.

## Supplementary Material


