# Supplementary material for: Mapping the evolution of acne research based on 100 top-cited articles: A bibliometric analysis of trends and hotspots from 2014 to 2023
Source: Medicine (Baltimore). 2024 May 24;103(21):e37657. doi: 10.1097/MD.0000000000037657 (PMC11124636; doi:10.1097/MD.0000000000037657)
Supplement: Supplementary file 1 [file medi-103-e37657-s001.docx]

**Supplementary Table S1** **List of the Top 100 Most Cited Papers in acne (2014–2023).**

| Ranking | Title | TC | ACY | *Journal* | First Author | Published year |
| --- | --- | --- | --- | --- | --- | --- |
| 1 | Guidelines of care for the management of acne vulgaris | 712 | 89.00 | *JOURNAL OF THE AMERICAN ACADEMY OF DERMATOLOGY* | Zaenglein, AL | 2016 |
| 2 | Propionibacterium acnes: from Commensal to Opportunistic Biofilm-Associated Implant Pathogen | 354 | 35.40 | *CLINICAL MICROBIOLOGY REVIEWS* | Achermann, Y | 2014 |
| 3 | A global perspective on the epidemiology of acne | 262 | 29.11 | *BRITISH JOURNAL OF DERMATOLOGY* | Tan, JKL | 2015 |
| 4 | Cutibacterium acnes (Propionibacterium acnes) and acne vulgaris: a brief look at the latest updates | 221 | 36.83 | *JOURNAL OF THE EUROPEAN ACADEMY OF DERMATOLOGY AND VENEREOLOGY* | Dreno, B | 2018 |
| 5 | Acne vulgaris | 195 | 21.67 | *NATURE REVIEWS DISEASE PRIMERS* | Tuchayi, SM | 2015 |
| 6 | Acne is an inflammatory disease and alterations of sebum composition initiate acne lesions | 164 | 16.40 | *JOURNAL OF THE EUROPEAN ACADEMY OF DERMATOLOGY AND VENEREOLOGY* | Zouboulis, CC | 2014 |
| 7 | Propionibacterium acnes: an update on its role in the pathogenesis of acne | 157 | 15.70 | *JOURNAL OF THE EUROPEAN ACADEMY OF DERMATOLOGY AND VENEREOLOGY* | Beylot, C | 2014 |
| 8 | What is new in the pathophysiology of acne, an overview | 154 | 22.00 | *JOURNAL OF THE EUROPEAN ACADEMY OF DERMATOLOGY AND VENEREOLOGY* | Dreno, B | 2017 |
| 9 | IL-1 beta Drives Inflammatory Responses to Propionibacterium acnes In Vitro and In Vivo | 154 | 15.40 | *JOURNAL OF INVESTIGATIVE DERMATOLOGY* | Kistowska, M | 2014 |
| 10 | European evidence-based (S3) guideline for the treatment of acne - update 2016-short version | 153 | 19.13 | *JOURNAL OF THE EUROPEAN ACADEMY OF DERMATOLOGY AND VENEREOLOGY* | Nast, A | 2016 |
| 11 | Cannabidiol exerts sebostatic and antiinflammatory effects on human sebocytes | 152 | 15.20 | *JOURNAL OF CLINICAL INVESTIGATION* | Olah, A | 2014 |
| 12 | Practical management of acne for clinicians: An international consensus from the Global Alliance to Improve Outcomes in Acne | 147 | 24.50 | *JOURNAL OF THE AMERICAN ACADEMY OF DERMATOLOGY* | Thiboutot, DM | 2018 |
| 13 | Systematic review of antibiotic resistance in acne: an increasing topical and oral threat | 131 | 16.38 | *LANCET INFECTIOUS DISEASES* | Walsh, TR | 2016 |
| 14 | Propionibacterium acnes Induces an IL-17 Response in Acne Vulgaris that Is Regulated by Vitamin A and Vitamin D | 129 | 12.90 | *JOURNAL OF INVESTIGATIVE DERMATOLOGY* | Agak, GW | 2014 |
| 15 | Adapalene loaded solid lipid nanoparticles gel: An effective approach for acne treatment | 126 | 12.60 | *COLLOIDS AND SURFACES B-BIOINTERFACES* | Jain, AK | 2014 |
| 16 | Propionibacterium acnes, an emerging pathogen: From acne to implant-infections, from phylotype to resistance | 125 | 12.50 | *MEDECINE ET MALADIES INFECTIEUSES* | Aubin, GG | 2014 |
| 17 | Propionibacterium acnes Induces IL-1 beta Secretion via the NLRP3 Inflammasome in Human Monocytes | 123 | 12.30 | *JOURNAL OF INVESTIGATIVE DERMATOLOGY* | Qin, M | 2014 |
| 18 | The balance of metagenomic elements shapes the skin microbiome in acne and health | 122 | 15.25 | *SCIENTIFIC REPORTS* | Barnard, E | 2016 |
| 19 | Host-microbiome interactions and recent progress into understanding the biology of acne vulgaris | 118 | 19.67 | *MICROBIOME* | O'Neill, AM | 2018 |
| 20 | Treatment Modalities for Acne | 118 | 14.75 | *MOLECULES* | Fox, L | 2016 |
| 21 | Anti-bacterial and anti-inflammatory properties of capric acid against Propionibacterium acnes: A comparative study with lauric acid | 108 | 10.80 | *JOURNAL OF DERMATOLOGICAL SCIENCE* | Huang, WC | 2014 |
| 22 | Potential Role of the Microbiome in Acne: A Comprehensive Review | 104 | 20.80 | *JOURNAL OF CLINICAL MEDICINE* | Lee, YB | 2019 |
| 23 | Use of a Picosecond Pulse Duration Laser With Specialized Optic for Treatment of Facial Acne Scarring | 104 | 11.56 | *JAMA DERMATOLOGY* | Brauer, JA | 2015 |

**Supplementary TABLE 1 (Continued).**

| Ranking | Title | TC | ACY | Journal | First Author | Published year |
| --- | --- | --- | --- | --- | --- | --- |
| 24 | Systematic review of the epidemiology of acne vulgaris | 103 | 25.75 | SCIENTIFIC REPORTS | Heng, AHS | 2020 |
| 25 | IL-17/Th17 Pathway Is Activated in Acne Lesions | 103 | 10.30 | PLOS ONE | Kelhala, HL | 2014 |
| 26 | Omega-3 Versus Omega-6 Polyunsaturated Fatty Acids in the Prevention and Treatment of Inflammatory Skin Diseases | 102 | 25.50 | INTERNATIONAL JOURNAL OF MOLECULAR SCIENCES | Balic, A | 2020 |
| 27 | Antagonism between Staphylococcus epidermidis and Propionibacterium acnes and its genomic basis | 98 | 12.25 | BMC GENOMICS | Christensen, GJM | 2016 |
| 28 | From pathogenesis of acne vulgaris to anti-acne agents | 95 | 19.00 | ARCHIVES OF DERMATOLOGICAL RESEARCH | Cong, TX | 2019 |
| 29 | Propionibacterium acnes Promotes Th17 and Th17/Th1 Responses in Acne Patients | 95 | 10.56 | JOURNAL OF INVESTIGATIVE DERMATOLOGY | Kistowska, M | 2015 |
| 30 | Autologous Platelet Rich Plasma: Topical Versus Intradermal After Fractional Ablative Carbon Dioxide Laser Treatment of Atrophic Acne Scars | 93 | 9.30 | DERMATOLOGIC SURGERY | Gawdat, HI | 2014 |
| 31 | Understanding innate immunity and inflammation in acne: implications for management | 89 | 9.89 | JOURNAL OF THE EUROPEAN ACADEMY OF DERMATOLOGY AND VENEREOLOGY | Dreno, B | 2015 |
| 32 | Propionibacterium acnes Activates the NLRP3 Inflammasome in Human Sebocytes | 89 | 8.90 | JOURNAL OF INVESTIGATIVE DERMATOLOGY | Li, ZJ | 2014 |
| 33 | Acne, the Skin Microbiome, and Antibiotic Treatment | 88 | 17.60 | AMERICAN JOURNAL OF CLINICAL DERMATOLOGY | Xu, HX | 2019 |
| 34 | Isotretinoin treatment for acne and risk of depression: A systematic review and meta-analysis | 88 | 12.57 | JOURNAL OF THE AMERICAN ACADEMY OF DERMATOLOGY | Huang, YC | 2017 |
| 35 | Skin microbiome and acne vulgaris: Staphylococcus, a new actor in acne | 86 | 12.29 | EXPERIMENTAL DERMATOLOGY | Dreno, B | 2017 |
| 36 | Acne Vulgaris | 82 | 13.67 | NEW ENGLAND JOURNAL OF MEDICINE | Zaenglein, AL | 2018 |
| 37 | Combined autologous platelet-rich plasma with microneedling verses microneedling with distilled water in the treatment of atrophic acne scars: a concurrent split-face study | 82 | 10.25 | JOURNAL OF COSMETIC DERMATOLOGY | Asif, M | 2016 |
| 38 | Efficacy and adverse events of oral isotretinoin for acne: a systematic review | 81 | 13.50 | BRITISH JOURNAL OF DERMATOLOGY | Vallerand, IA | 2018 |
| 39 | Vitamin B-12 modulates the transcriptome of the skin microbiota in acne pathogenesis | 81 | 9.00 | SCIENCE TRANSLATIONAL MEDICINE | Kang, DZ | 2015 |
| 40 | From new findings in acne pathogenesis to new approaches in treatment | 80 | 8.89 | JOURNAL OF THE EUROPEAN ACADEMY OF DERMATOLOGY AND VENEREOLOGY | Gollnick, HPM | 2015 |
| 41 | Skin expression of mammalian target of rapamycin and forkhead box transcription factor O1, and serum insulin-like growth factor-1 in patients with acne vulgaris and their relationship with diet | 79 | 9.88 | BRITISH JOURNAL OF DERMATOLOGY | Agamia, NF | 2016 |
| 42 | Acne and quality of life - impact and management | 79 | 8.78 | JOURNAL OF THE EUROPEAN ACADEMY OF DERMATOLOGY AND VENEREOLOGY | Gieler, U | 2015 |
| 43 | Why Topical Retinoids Are Mainstay of Therapy for Acne | 78 | 11.14 | DERMATOLOGY AND THERAPY | Leyden, J | 2017 |
| 44 | Recent Advances in Acne Pathogenesis: Implications for Therapy | 78 | 7.80 | AMERICAN JOURNAL OF CLINICAL DERMATOLOGY | Das, S | 2014 |
| 45 | ROS-Responsive Microneedle Patch for Acne Vulgaris Treatment | 76 | 12.67 | ADVANCED THERAPEUTICS | Zhang, YQ | 2018 |

**Supplementary TABLE 1 (Continued).**

| Ranking | Title | TC | ACY | Journal | First Author | Published year |
| --- | --- | --- | --- | --- | --- | --- |
| 46 | Acne prevalence and associations with lifestyle: a cross-sectional online survey of adolescents/young adults in 7 European countries | 75 | 12.50 | JOURNAL OF THE EUROPEAN ACADEMY OF DERMATOLOGY AND VENEREOLOGY | Wolkenstein, P | 2018 |
| 47 | Acne vulgaris: The metabolic syndrome of the pilosebaceous follicle | 71 | 11.83 | CLINICS IN DERMATOLOGY | Melnik, BC | 2018 |
| 48 | Large-scale international study enhances understanding of an emerging acne population: adult females | 71 | 7.89 | JOURNAL OF THE EUROPEAN ACADEMY OF DERMATOLOGY AND VENEREOLOGY | Dreno, B | 2015 |
| 49 | Antibiotic stewardship in dermatology: limiting antibiotic use in acne | 71 | 7.10 | EUROPEAN JOURNAL OF DERMATOLOGY | Dreno, B | 2014 |
| 50 | Adverse effects of isotretinoin: A large, retrospective review | 70 | 10.00 | DERMATOLOGIC THERAPY | Brzezinski, P | 2017 |
| 51 | Laboratory Monitoring During Isotretinoin Therapy for Acne A Systematic Review and Meta-analysis | 70 | 8.75 | JAMA DERMATOLOGY | Lee, YH | 2016 |
| 52 | Propionibacterium acnes and Acne Vulgaris: New Insights from the Integration of Population Genetic, Multi-Omic, Biochemical and Host-Microbe Studies | 69 | 13.80 | MICROORGANISMS | McLaughlin, J | 2019 |
| 53 | What's new in the physiopathology of acne? | 69 | 7.67 | BRITISH JOURNAL OF DERMATOLOGY | Suh, DH | 2015 |
| 54 | Insulin and insulin-like growth factor-1 can modulate the phosphoinositide-3-kinase/Akt/FoxO1 pathway in SZ95 sebocytes in vitro | 68 | 7.56 | MOLECULAR AND CELLULAR ENDOCRINOLOGY | Mirdamadi, Y | 2015 |
| 55 | Propionibacterium acnes and antimicrobial resistance in acne | 67 | 9.57 | CLINICS IN DERMATOLOGY | Dessinioti, C | 2017 |
| 56 | A consensus-based practical and daily guide for the treatment of acne patients | 67 | 8.38 | JOURNAL OF THE EUROPEAN ACADEMY OF DERMATOLOGY AND VENEREOLOGY | Gollnick, HP | 2016 |
| 57 | Staphylococcal LTA-Induced miR-143 Inhibits Propionibacterium acnes-Mediated Inflammatory Response in Skin | 67 | 8.38 | JOURNAL OF INVESTIGATIVE DERMATOLOGY | Xia, XL | 2016 |
| 58 | Antibiotic Resistance in Acne Treatment | 65 | 9.29 | JAMA DERMATOLOGY | Adler, BL | 2017 |
| 59 | Mechanistic target of rapamycin (mTOR) expression is increased in acne patients' skin | 65 | 8.13 | EXPERIMENTAL DERMATOLOGY | Monfrecola, G | 2016 |
| 60 | Acne vulgaris and risk of depression and anxiety: A meta-analytic review | 64 | 16.00 | JOURNAL OF THE AMERICAN ACADEMY OF DERMATOLOGY | Samuels, DV | 2020 |
| 61 | Different Propionibacterium acnes Phylotypes Induce Distinct Immune Responses and Express Unique Surface and Secreted Proteomes | 64 | 8.00 | JOURNAL OF INVESTIGATIVE DERMATOLOGY | Yu, Y | 2016 |
| 62 | The Psychosocial Impact of Acne Vulgaris | 64 | 8.00 | INDIAN JOURNAL OF DERMATOLOGY | Hazarika, N | 2016 |
| 63 | Acne Vulgaris: Diagnosis and Treatment | 62 | 12.40 | AMERICAN FAMILY PHYSICIAN | Oge, LK | 2019 |
| 64 | Homeostasis of the sebaceous gland and mechanisms of acne pathogenesis | 62 | 12.40 | BRITISH JOURNAL OF DERMATOLOGY | Clayton, RW | 2019 |
| 65 | Randomized phase 3 evaluation of trifarotene 50 mu g/g cream treatment of moderate facial and truncal acne | 62 | 12.40 | JOURNAL OF THE AMERICAN ACADEMY OF DERMATOLOGY | Tan, J | 2019 |
| 66 | Staphylococcus epidermidis: A Potential New Player in the Physiopathology of Acne? | 62 | 12.40 | DERMATOLOGY | Claudel, JP | 2019 |
| 67 | Acne vulgaris, probiotics and the gut-brain-skin axis: from anecdote to translational medicine | 62 | 6.20 | BENEFICIAL MICROBES | Bowe, WP | 2014 |

**Supplementary TABLE 1 (Continued).**

| Ranking | Title | TC | ACY | Journal | First Author | Published year |
| --- | --- | --- | --- | --- | --- | --- |
| 68 | In vitro antibacterial and synergistic effect of phlorotannins isolated from edible brown seaweed Eisenia bicyclis against acne-related bacteria | 62 | 6.20 | ALGAE | Lee, JH | 2014 |
| 69 | Low Usefulness of Potassium Monitoring Among Healthy Young Women Taking Spironolactone for Acne | 61 | 6.78 | JAMA DERMATOLOGY | Plovanich, M | 2015 |
| 70 | Acne Treatment Based on Selective Photothermolysis of Sebaceous Follicles with Topically Delivered Light-Absorbing Gold Microparticles | 61 | 6.78 | JOURNAL OF INVESTIGATIVE DERMATOLOGY | Paithankar, DY | 2015 |
| 71 | Efficacy and Safety of Topical Clascoterone Cream, 1%, for Treatment in Patients With Facial Acne Two Phase 3 Randomized Clinical Trials | 60 | 15.00 | JAMA DERMATOLOGY | Hebert, A | 2020 |
| 72 | Microneedling for acne scars in Asian skin type: an effective low cost treatment modality | 60 | 6.00 | JOURNAL OF COSMETIC DERMATOLOGY | Dogra, S | 2014 |
| 73 | The influence of exposome on acne | 59 | 9.83 | JOURNAL OF THE EUROPEAN ACADEMY OF DERMATOLOGY AND VENEREOLOGY | Dreno, B | 2018 |
| 74 | Treatment of acne with tea tree oil (melaleuca) products: A review of efficacy, tolerability and potential modes of action | 59 | 6.56 | INTERNATIONAL JOURNAL OF ANTIMICROBIAL AGENTS | Hammer, KA | 2015 |
| 75 | Platelet-Rich Plasma Versus CROSS Technique With 100% Trichloroacetic Acid Versus Combined Skin Needling and Platelet Rich Plasma in the Treatment of Atrophic Acne Scars: A Comparative Study | 59 | 5.90 | DERMATOLOGIC SURGERY | Nofal, E | 2014 |
| 76 | The Skin Microbiome: A New Actor in Inflammatory Acne | 58 | 14.50 | AMERICAN JOURNAL OF CLINICAL DERMATOLOGY | Dreno, B | 2020 |
| 77 | New Insights into Acne Pathogenesis: Propionibacterium Acnes Activates the Inflammasome | 58 | 5.80 | JOURNAL OF INVESTIGATIVE DERMATOLOGY | Contassot, E | 2014 |
| 78 | Azelaic Acid: Properties and Mode of Action | 58 | 5.80 | SKIN PHARMACOLOGY AND PHYSIOLOGY | Sieber, MA | 2014 |
| 79 | A review of the role of sebum in the mechanism of acne pathogenesis | 57 | 8.14 | JOURNAL OF COSMETIC DERMATOLOGY | Li, XC | 2017 |
| 80 | Sex hormones and acne | 57 | 8.14 | CLINICS IN DERMATOLOGY | Ju, Q | 2017 |
| 81 | Relationship between the severity of acne vulgaris and antimicrobial resistance of bacteria isolated from acne lesions in a hospital in Japan | 57 | 5.70 | JOURNAL OF MEDICAL MICROBIOLOGY | Nakase, K | 2014 |
| 82 | Differential effectiveness of selected non-psychotropic phytocannabinoids on human sebocyte functions implicates their introduction in dry/seborrhoeic skin and acne treatment | 56 | 7.00 | EXPERIMENTAL DERMATOLOGY | Olah, A | 2016 |
| 83 | Phenotype and Antimicrobial Activity of Th17 Cells Induced by Propionibacterium acnes Strains Associated with Healthy and Acne Skin | 55 | 9.17 | JOURNAL OF INVESTIGATIVE DERMATOLOGY | Agak, GW | 2018 |
| 84 | Acne as a chronic systemic disease | 55 | 5.50 | CLINICS IN DERMATOLOGY | Zouboulis, CC | 2014 |
| 85 | Management of Acne Vulgaris | 54 | 18.00 | JAMA-JOURNAL OF THE AMERICAN MEDICAL ASSOCIATION | Eichenfield, DZ | 2021 |
| 86 | Approaches to limit systemic antibiotic use in acne: Systemic alternatives, emerging topical therapies, dietary modification, and laser and light-based treatments | 54 | 10.80 | JOURNAL OF THE AMERICAN ACADEMY OF DERMATOLOGY | Barbieri, JS | 2019 |

**Supplementary TABLE 1 (Continued).**

| Ranking | Title | TC | ACY | Journal | First Author | Published year |
| --- | --- | --- | --- | --- | --- | --- |
| 87 | Prevalence and Risk Factors of Acne Scarring Among Patients Consulting Dermatologists in the Unites States | 54 | 7.71 | JOURNAL OF DRUGS IN DERMATOLOGY | Tan, J | 2017 |
| 88 | Oral Spironolactone for Acne Vulgaris in Adult Females: A Hybrid Systematic Review | 53 | 7.57 | AMERICAN JOURNAL OF CLINICAL DERMATOLOGY | Layton, AM | 2017 |
| 89 | Dietary glycemic factors, insulin resistance, and adiponectin levels in acne vulgaris | 53 | 6.63 | JOURNAL OF THE AMERICAN ACADEMY OF DERMATOLOGY | Cerman, AA | 2016 |
| 90 | A synergistic approach of adapalene-loaded nanostructured lipid carriers, and vitamin C co-administration for treating acne | 53 | 6.63 | DRUG DEVELOPMENT AND INDUSTRIAL PHARMACY | Jain, A | 2016 |
| 91 | Supplementation with Lactobacillus rhamnosus SP1 normalises skin expression of genes implicated in insulin signalling and improves adult acne | 53 | 6.63 | BENEFICIAL MICROBES | Fabbrocini, G | 2016 |
| 92 | Effect of Dietary Supplementation with Omega-3 Fatty Acid and Gamma-linolenic Acid on Acne Vulgaris: A Randomised, Double-blind, Controlled Trial | 53 | 5.30 | ACTA DERMATO-VENEREOLOGICA | Jung, JY | 2014 |
| 93 | Increased flare of acne caused bylong-timemask wearing during COVID-19 pandemic among general population | 52 | 13.00 | DERMATOLOGIC THERAPY | Han, CX | 2020 |
| 94 | Microbiological Profile of Sarecycline, a Novel Targeted Spectrum Tetracycline for the Treatment of Acne Vulgaris | 52 | 10.40 | ANTIMICROBIAL AGENTS AND CHEMOTHERAPY | Zhanel, G | 2019 |
| 95 | COVID-19 related masks increase severity of both acne (maskne) and rosacea (mask rosacea): Multi-center, real-life, telemedical, and observational prospective study | 51 | 17.00 | DERMATOLOGIC THERAPY | Damiani, G | 2021 |
| 96 | Skin barrier and microbiome in acne | 51 | 8.50 | ARCHIVES OF DERMATOLOGICAL RESEARCH | Rocha, MA | 2018 |
| 97 | Comparative study on efficacy and safety of 1550nm Er:Glass fractional laser and fractional radiofrequency microneedle device for facial atrophic acne scar | 51 | 5.67 | JOURNAL OF COSMETIC DERMATOLOGY | Chae, WS | 2015 |
| 98 | Relationships of Self-Reported Dietary Factors and Perceived Acne Severity in a Cohort of New York Young Adults | 51 | 5.10 | JOURNAL OF THE ACADEMY OF NUTRITION AND DIETETICS | Burris, J | 2014 |
| 99 | IL-36 cytokines are increased in acne and hidradenitis suppurativa | 50 | 7.14 | ARCHIVES OF DERMATOLOGICAL RESEARCH | Di Caprio, R | 2017 |
| 100 | The Role of Photodynamic Therapy in Acne: An Evidence-Based Review | 50 | 7.14 | AMERICAN JOURNAL OF CLINICAL DERMATOLOGY | Boen, M | 2017 |

Abbreviations: TC, total citations; ACY. average citations per year.
